# The effects of pramipexole on motivational vigour during a saccade task: a placebo-controlled study in healthy adults

**DOI:** 10.1007/s00213-024-06567-z

**Published:** 2024-03-18

**Authors:** Sheena K. Au-Yeung, Don Chamith Halahakoon, Alexander Kaltenboeck, Philip Cowen, Michael Browning, Sanjay G Manohar

**Affiliations:** 1 Department of Psychiatry, University of Oxford, Oxford, OX3 7JX UK; 2 Clinical Psychology Unit, University of Sheffield, Cathedral Court Floor F 1 Vicar Lane, Sheffield, S1 2LT UK; 3 Oxford Health NHS Foundation Trust, Warneford Hospital, Oxford, OX3 7JX UK; 4 Clinical Division of Social Psychiatry, Department of Psychiatry and Psychotherapy, Medical University of Vienna, Vienna General Hospital, Vienna, 1090 Austria; 5 Nuffield Department of Clinical Neurosciences, John Radcliffe Hospital, University of Oxford, Level 6 West Wing, Oxford, OX3 9DU UK

**Keywords:** Pramipexole, Motivation, Vigour, Reward, Eye movement, Saccade, Dopamine, Eye tracking

## Abstract

Motivation allows us to energise actions when we expect reward and is reduced in depression. This effect, termed motivational vigour, has been proposed to rely on central dopamine, with dopaminergic agents showing promise in the treatment of depression. This suggests that dopaminergic agents might act to reduce depression by increasing the effects of reward or by helping energise actions. The aim of the current study was to investigate whether the dopamine agonist pramipexole enhanced motivational vigour during a rewarded saccade task. In addition, we asked whether the effects of pramipexole on vigour differ between reward contingent on performance and guaranteed reward. Healthy adult participants were randomised to receive either pramipexole (*n* = 19) or placebo (controls *n* = 18) for 18 days. The vigour of saccades was measured twice, once before the administration of study medication (Time 1) and after taking it for 12–15 days (Time 2). To separate motivation by contingency vs. reward, saccadic vigour was separately measured when (1) rewards were contingent on performance (2) delivered randomly with matched frequency, (3) when reward was guaranteed, (4) when reward was not present at all. Motivation increased response vigour, as expected. Relative to placebo, pramipexole also increased response vigour. However, there was no interaction, meaning that the effects of reward were not modulated by drug, and there was no differential drug effect on contingent vs. guaranteed rewards. The effect of pramipexole on vigour could not be explained by a speed/accuracy trade-off, nor by autonomic arousal as indexed by pupillary dilation. Chronic D2 stimulation increases general vigour, energising movements in healthy adults irrespective of extrinsic reward.

**Supplementary Information:**

The online version contains supplementary material available at 10.1007/s00213-024-06567-z.

## Introduction

Anhedonia is defined as a loss of interest or pleasure, and is recognised as one of the core symptoms of depression (The Diagnostic and Statistical Manual of Mental Disorders 5th ed. [DSM–5]; American Psychiatric Association, 2013)American Psychological Association 2013). In people with depression, anhedonia is associated with reduced motivation to exert both physical and cognitive effort to obtain rewards (Horne et al. 2021). Response vigour, one measure of motivation, is thought to be mediated by dopamine, a neurotransmitter typically known for its role in reward learning. Motivation vigour is characterised by faster responses in relation to reward. Previous models (Niv et al. 2007) have posited that tonic dopamine signals encode average reward rate which influence motivational vigour. Accordingly, response vigour tracks manipulations of average reward rate, and boosting dopamine levels in the brain amplifies the strength of this relationship (Beierholm et al. 2013). In contrast, phasic dopamine signals are thought to signify reward prediction error and are also associated with vigour after cues predictive of higher reward. Disruption in dopaminergic reward networks is thought to underlie motivational deficits in people with anhedonia and depression (Höflich et al. 2019).

Standard antidepressant treatments, such as selective serotonin reuptake inhibitors, are thought to be relatively ineffective at improving the reward related dysfunction of anhedonia (Höflich et al. 2019). Given the ability of dopamine to modulate motivational vigour, dopaminergic medications have been noted as a potential treatment for depression that may be particularly effective in the treatment of anhedonia (Shelton and Tomarken 2001). Pramipexole is a dopamine agonist that is licensed for treating Parkinson’s Disease and Restless leg Syndrome. It acts selectively at dopamine receptors within the D2 receptor subfamily (includes D2, D3, D4 receptors subtypes) with particularly high affinity for the D3 subtype (Dooley and Markham 1998). There is preliminary clinical evidence showing that pramipexole may be effective for treating depression (Tundo et al. 2019), however the mechanisms by which it exerts this clinical effect, are unclear.

One possibility is that pramipexole improves symptoms of depression by increasing reward processing and response vigour. However, previous studies in humans looking at the cognitive effect of pramipexole suggest that it disrupts, rather than enhances, reward processing, at least in the short term. Specifically, a single dose of pramipexole leads to reduced accuracy on stimulus-response tasks (Gallant et al. 2016), impaired reward learning (Pizzagalli et al. 2008), larger feedback related negativities, and reduced activation in the dorsal anterior cingulate region of the brain (associated with blunted reward learning) that is thought to be responsible for the integration of reinforcement histories across time (Santesso et al. 2009). In rat studies, paradoxical effect of acute pramipexole is thought to be caused by an initial effect of the drug on inhibitory pre-synaptic D2-like receptors which inhibits dopamine release. Prolonged treatment schedules (i.e. 1–2 weeks), produce downregulation of the pre-synaptic receptors, and the emergence of the expected, post-synaptic effects of the drug (Chernoloz et al. 2009, 2012).

This suggests that increased dopamine transmission after longer term pramipexole treatment is required to produce the desired clinical effect for people with depression. Interestingly, a recent study by Whitton et al. (Whitton et al. 2020) showed that 6 weeks administration of pramipexole in people with major depressive disorder significantly improved depressive symptoms, but no change in response bias, reward sensitivity or learning rate from pre- to post-treatment in a probabilistic reward task. In contrast, 12 weeks treatment of pramipexole or ropinirole have been shown to remediate reward processing in a feedback based probabilistic classification task in patients with Parkinson’s disease with selective deficits in reward processing (Bodi et al. 2009). These varied findings raise questions as to how pramipexole may work, and how it influences reward processing in general and motivational vigour in particular.

Energisation of movement provides a precise way to measure motivation (Takikawa et al. 2002; Reppert et al. 2015). When a rewarding incentive is offered, eye movements to a target speed up, more than would be expected for the size of the movement (Manohar et al. 2015). Such increases in speed have been termed *vigour*, and are coupled to dopaminergic transmission, motivation and reward expectation (Niv et al. 2007; Beierholm et al. 2013). Vigour can be measured in an incentivised saccade task(Manohar et al. 2017), where a cue that indicates reward is available can increase the speed of subsequent eye movements towards a visual target. The controllability or contingency of rewards on offer may be crucial. Movements are faster if reward is *guaranteed*, compared to when no reward is available. But movements are even faster when reward will depend on performance (*contingent* reward), and reward expectation and contingency seem to contribute independently to vigour(Manohar et al. 2017). In Parkinson’s disease, dopaminergic stimulation increases motivation by contingent reward, but in contrast, weakens motivation by guaranteed reward(Grogan et al. 2020). One hypothesis to explain this is that phasic dopamine may signal contingency, and is attenuated in patients, but can be restored with levodopa. However, those observations were based on a single-dose manipulation, which may work in a fundamentally different way to longer-term treatment. Moreover, people with depression generally have an intact dopamine system, and it is not clear what the effect of a D2 agonist would be in this case. It is possible that D2 stimulation might specifically increase vigour when reward is contingent on performance.

An alternative hypothesis is that pramipexole may have a more general effect on oculomotor response irrespective of motivation by extrinsic reward. Faster movements carry a cost which is traded against background intrinsic motivation (“motor motivation”), which may determine movement speed *independent* of extrinsic reward (Mazzoni et al. 2007). Changes in motor motivation linked to deficiency in striatal dopamine may provide a common mechanism for bradykinesia (movement slowing) in Parkinson’s Disease, and psychomotor disturbances in depression (Liberg and Rahm 2015).

The current study aimed to investigate whether two weeks treatment with the dopamine agonist pramipexole alters, relative to placebo, the two types of motivational vigour in healthy adults. The saccade task (Manohar et al. 2017) was used as the measure of motivational vigour in response to cues predictive of reward. We asked whether motivation vigour is by contingent and guaranteed rewards are differentially affected or whether it simply increase motor vigour regardless of rewards. We further ask whether any increase in vigour due to pramipexole could be due to a trade-off between speed and accuracy, or to changes in autonomic arousal as indexed by pupil dilatation.

## Method

### Participants

Forty-two participants took part in the study. Participants were included in if they were male or female, age between 18 and 45, in good physical and mental health, and were able to give informed consent. Participant had sufficient knowledge of English to understand and complete study tasks, were willing to refrain from driving, cycling, or operating heavy machinery if necessary, and willing to refrain from drinking while taking part in the study. Participant were not able to enter the study if any of the following applied: current or past psychiatric disorder; first-degree relative with a diagnosis of schizophrenia-spectrum or other psychotic, or bipolar disorder; Body Mass Index below 18 or above 30; history of unexplained hallucinations or impulse control problems (e.g. pathological gambling); any contraindication to pramipexole; lactose intolerance; any current or past physical illness that has the potential to significantly affect mental functioning (e.g. epilepsy, hypothyroidism, Parkinson’s disease, multiple sclerosis etc.); were pregnant, or lactating; sexually active woman who were not using a medically accepted method of contraception; current or previous intake (last three months) of any medication that has a significant potential to affect mental functioning; any intake of recreational drugs in the last 3 months; regular alcohol consumption of more than 14 units a week or excessive alcohol consumption up to three days before the experiment; regular smoker (> 5 cigarettes per day); excessive caffeine user (> 6 caffeinated drinks per day); previous participation in a study using the same or similar tasks.

### Materials

#### Eye-tracking task

**Fig. 1:**
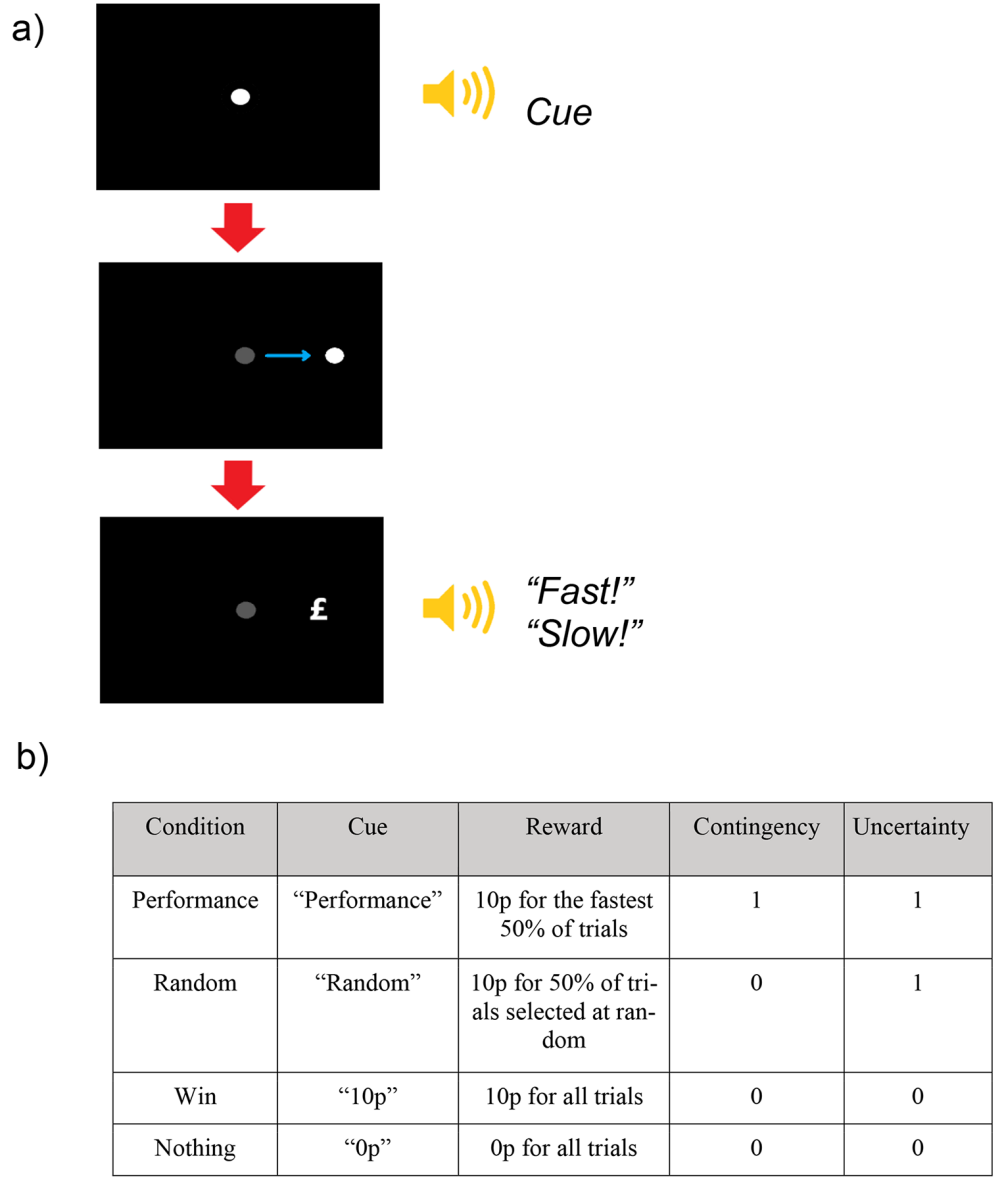
**(a)** Illustration of trial sequence. For each trial, participant fixated a disc at the centre of the screen. They then hear one of four cues indicating how reward will be determined for that trial. A target then appears to the left or right of the centre target. Participants then move their eyes towards the target. Once the target is fixated, participant received feedback about the amount of reward won and their speed. **(b)** experimental manipulation of the four conditions

Eye movements were recorded using an Eyelink 1000 eye tracker (SR Research) with a viewing distance of 70 cm. Participants’ head position was fixed using a desktop mount headrest. The experimental paradigm is an adapted saccade task identical to one used in the first experiment of a previous study by Manohar et al. (Manohar et al. 2017). Participants were asked to move their eyes as fast as possible to look at a target which appears either on the left or right side of the screen. At the start of each trial, participants fixated a central disc, and after 500ms of fixation, heard one of four spoken cues that indicated how the reward would be determined. After a randomly selected interval between 1400-1600ms, a target disc appeared, randomly chosen to appear 11 degrees to the left or right of fixation. Participants had to perform a horizontal saccade and fixate the target as quickly as possible. Once their gaze arrived at the target, participants were given feedback about their speed (either “*Fast*” or “*Slow*”), and about whether they received a reward. Three hundred and eighty-four trials (96 trials in each condition) from four different cued conditions were intermixed. In the Performance-based-gain condition (cue: “performance”), the fastest 50% of reaction times were rewarded with 10 pence, using an adaptive median split on response time. In the random-gain condition (cue: “random”), 50% of their responses were randomly rewarded with 10 pence. Therefore, these two conditions were matched for expected reward, and uncertainty, but differed only in contingency on speed of performance. In the non-contingent “10 pence” gain condition (“Win” condition), reward was guaranteed regardless of speed of performance. In the 0 pence gained condition (“Nothing” condition), no rewards were given. These two conditions were therefore matched in having no uncertainty or contingency on speed of performance, but differed only in expected reward (see Fig. 1). The Win and Nothing conditions therefore tests whether motivation vigour is affected by the mere presence and absence of rewards respectively. In these conditions, the outcomes were “guaranteed” in the sense that the experiment always waited until a saccade was made and a reward was delivered. “Fast” and “Slow” feedback, as well as the reward in the contingent condition, were determined on each trial relative to that participant’s median RT in that condition over the last 20 trials (or fewer at the beginning of the experiment), to equate conditions and participants for the amount of reward received.

### Procedure

Participants were recruited by word of mouth, emails to departmental and college mailing lists at the University of Oxford, online and print advertisements (including social networks like Twitter) and posters located in colleges and university departments. Participants attended a screening visit where their eligibility was assessed by a medical doctor (DH or AK) prior to their inclusion in the study. The screening visit consisted of an explanation of study procedures, collection of written informed consent, completion of the Structured Clinical Interview of the DSM-5 (SCID-5, First et al. 2015) and Beck’s Depression Inventory (Beck et al. 1996) to rule out psychiatric conditions and depression, and other questionnaires and behavioural tasks not reported here as part of a wider research programme which was pre-registered at https://clinicaltrials.gov/ct2/show/NCT03681509 (For other published studies from the programme, see Martens et al. 2021; Halahakoon et al. 2022; Kaltenboeck et al. 2022). The maximum duration of time allowed between screening and inclusion in the study was 28 days.

**Fig. 2:**
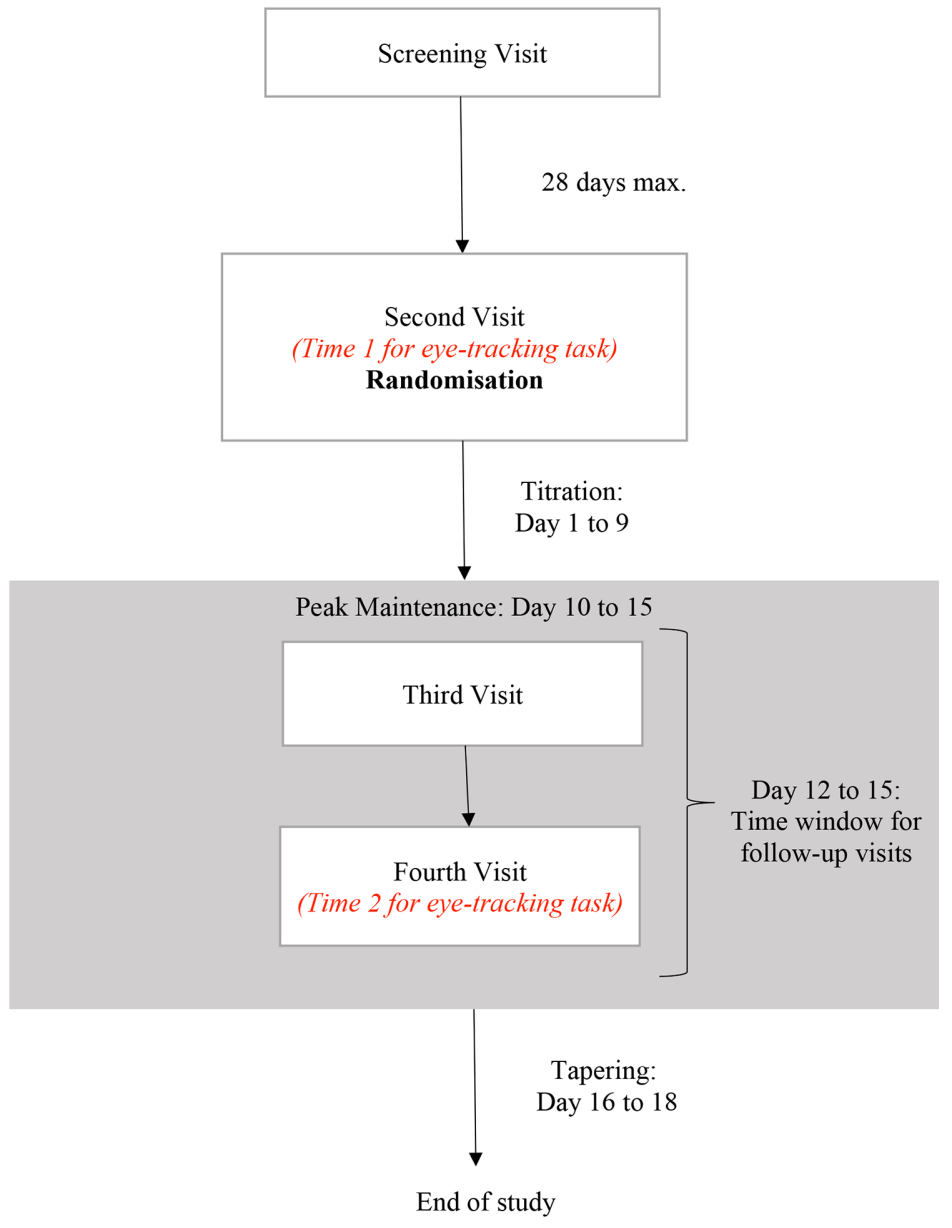
Study Timeline. Participants attended the screening visit and completed eligibility assessment. If eligible, they were invited to attend the second visit no more than 28 days after the screening visit. Participant completed the eye-tracking task for the first time during the second visit. After completion the baseline measurement, they were randomized to receive either pramipexole or placebo and took their first dose of the assigned medication at the visit. Pramipexole was initiated at a dose of 0.25 mg/day of pramipexole salt and was increased by 0.25 mg every three days to a maximum of 1 mg/day (achieved on day 10). Participants continue to maintain 1 mg dose between day 10 and 15. The third and fourth follow-up visits are completed within the day 12 to 15 time window. Participants were then tapered off the medication by reducing the dose by 0.25 mg per day over 3 days between day 16 to 18

If a participant was deemed eligible to participate in the study during screening, they were invited to three further research visits in total to complete a range of tasks as part of a wider study on the effects of pramipexole on emotional and reward information processing. Here we will only describe two of the visits (the second and fourth visit) that are relevant to the current experiment (see Fig. 2 for study timeline).

#### Randomisation and Intervention

Participants were randomised in a 1:1 ratio to receive a 15-day course of pramipexole or placebo which were encapsulated in identical capsules. Randomisation was stratified by gender, with both the study team and participant blind to allocated group. Given that our participants were healthy volunteers, a lower dosage and shorter period of drug administration were selected than for people with depression requiring treatment. A lower dose also reduces the chance of unblinding early due to adverse effects. Pramipexole was initiated at a dose of 0.25 mg/day of pramipexole salt and was increased by 0.25 mg every three days to a maximum of 1 mg/day (achieved on day 10). Following this, participants continued 1 mg/day until testing was finished (day 15). Participants then gradually reduced and stopped the pramipexole over the next 3 days. The apparent dose of the placebo group (i.e. the number of capsules taken per day) was increased identically to the pramipexole group. Participants were provided with a pill box with the correct dosage of medication prepared for each day. The pill box was returned at the end of the study. Telephone calls were also conducted by the medics (author DH and AK) on Day 2 and Day 8 of the titration period to ensure that participants were taking the medication correctly and to monitor side effects.

#### Second Visit (Treatment day 1, referred to as ***Time 1*** hereafter)

Baseline self-report questionnaires were collected. After that, baseline measures in a battery of emotion and reward related behavioural cognitive tasks, including the eye tracking task reported here, were collected. Participants were rewarded with small amount of extra payment depending on their task performance. Participants were provided with study medication which they took for the next 15 days.

#### Fourth Visit (between treatment days 12 to 15, referred to as ***Time 2*** hereafter)

Further questionnaires and behavioural measurements were taken. Participants repeated the battery of computerised cognitive tasks measuring different aspects of reward and emotion related information processing, including the eye tracking and state questionnaire measures.

### Data analysis

The data was analysed by the first author who was not involved in the design and data collection of the study, but was not blinded to group medication status. Out of the 42 participants, two participants from the placebo group dropped out of the study due to side effects and were therefore excluded from the analysis. All remaining participants reached the final dosage of 1 mg. One participant’s data was lost due to equipment failure and two further participants were excluded due to their main sequence (relationship between saccadic velocity and amplitude (Bahill et al. 1975) being two standard deviations away from the mean of the population. The main outcome of interest is calculated as deviance from this sequence (see below). Therefore, the final sample consists of 37 participants (controls *n* = 18, pramipexole *n* = 19). Participant group characteristics are reported in Table 1. The two groups of participants did not differ in age, years in education, or depression symptoms (both at screening and during the two testing sessions). For the included participants, analysis followed that of previous studies (Manohar et al. 2017; Grogan et al. 2020). The experiment was gaze contingent such that a trial could not begin until central fixation was established for 500ms, and the eye had to land on the target on every trial for the experiment to progress. The first saccade larger than 1 degree was analysed. Trials were excluded from analysis if (1) the saccade’s amplitude was smaller than 1^o^ or greater than 20^o^, (2) reaction times were less than 100ms or greater than 500ms, (3) saccadic velocity was greater than 1000 deg/s, (4) a blink was present, or (5) the angle between landing position of the saccade and the target from the central fixation point was greater than 30^o^. On average, each participant had 26.8 out of 384 trials excluded (6.97%), ranging from 0 to 117 trials.

**Table 1:** Participant group characteristics

	Control	Pramipexole	*p*-value
*n*	18	19	
Gender			
*Male*	10	9	
*Female*	8	10	
Mean Age (*SD*)	24.5 (7.13)	22.68 (3.76)	0.345
BDI Screening	2.61 (4.03)	1.68 (1.73)	0.378
BDI Time 1	3.00 (3.35)	2.11 (2.60)	0.375
BDI Time 2	2.78 (3.41)	3.11 (3.46)	0.774
Years in Education	17.3 (3.12)	17.1 (2.83)	0.817

*Note. Independent sample t-tests were conducted to examine between-group differences in age and BDI scores.*

Our primary dependent variable was saccadic vigour. This was indexed by the *Velocity Residuals*, i.e. how much faster or slower the saccade is compared to the average velocity of a saccade of the same size (Manohar et al. 2017). Peak saccadic velocity is first calculated for the first saccade after the target onset in degrees per second (^o^/s). Then, the effect of saccadic amplitude is regressed out from peak velocity across conditions and across both sessions, separately for each participant, resulting in velocity residuals. Note that residuals were normalised by pooling trials across both sessions for each participant, then fixing the main sequence, and then calculating residuals for each session separately. This mean that if some participants are much faster in Time 2, their residuals at Time 1 will appear lower (due to the normalisation across sessions). This normalisation is important because every person’s eye has different mechano-physiological properties and therefore a different main sequence.

We fitted a linear mixed effects (LME) model to all trials using SPSS Version 27 on four eye movement measures. There were four fixed effects variables: 2 Time (Time 1 vs. Time 2) x 2 Group (Pramipexole vs. Control) x 2 Motivation (High vs. Low) x Contingency (Contingent vs. Guaranteed). Fixed effects including all main effects of each variable and all interaction effects between variables were computed in the model. We included all random effects per subject with a compound symmetry covariance, i.e. repeated measures design. Post-hoc contrasts were performed when interactions indicated.

Four secondary dependent variables were also investigated: (1) *Saccadic Reaction Time (RT) is the time between target onset and saccade onset in milliseconds (ms). Saccade onset time was calculated using the Eyelink saccade parser which uses standard combined velocity and acceleration criteria*; (2) *Saccadic Amplitude* is the size of the saccade in degrees (^o^); (3) *Saccadic Endpoint Variability* is the standard error of saccade amplitude, i.e. the standard deviation of saccade amplitude across trials of the same time points and condition type for each participant; (4) Pupil Dilatation is the change in pupil size from cue onset to before target onset (a.u.). Saccadic RT, amplitude, and pupil dilatation were analysed in the same way as velocity residuals using LME. For Saccadic Endpoint Variability, a four-way ANOVA was computed because standard deviations were calculated across trials within each condition, for each time point for each participant.

## Results

### Velocity residuals

**Fig. 3:**
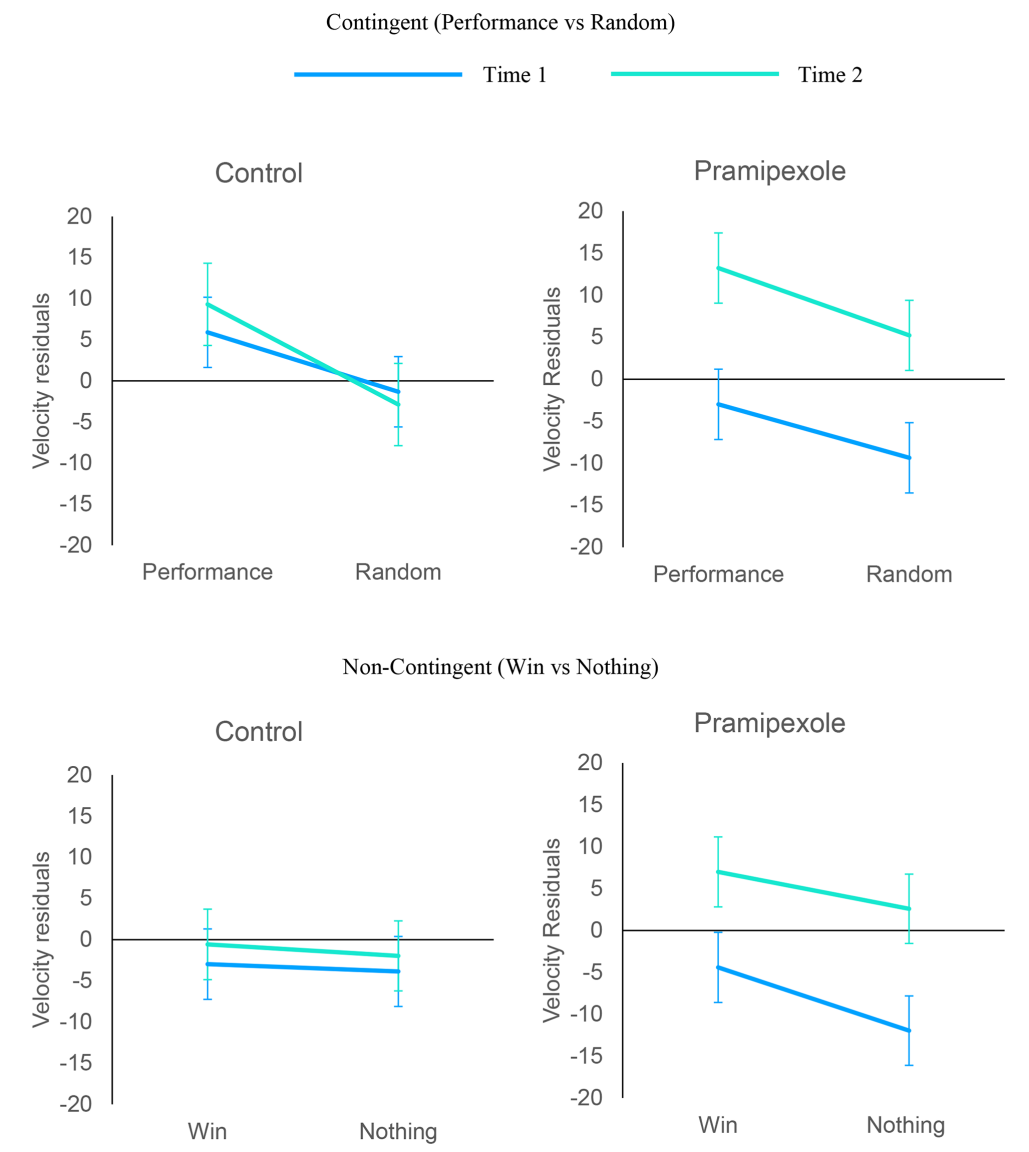
Velocity residuals. Error bars represents +/- 1 SEM

Motivation increased vigour as measured by the velocity residuals (Fig. 3; *F*[1, 5.09] = 5.28, *p* = .022). Crucially, *v*igour was also increased by pramipexole, (Time × Group interaction, (*F*[1, 511] = 5.87, *p* = .015. There was also a practice effect (main effect of Time, *F*(1, 511) = 9.06, *p* = .003). None of the other fixed effects were significant (all *p*s > 0.05).

To further examine the drug effect (group × time), LME models were fitted separately for the pramipexole and control group. For the pramipexole group, there was a significant effect of time, *F*(1, 102) = 21.6, *p* = .001, indicating increased vigour from Time 1 (*M* = -7.18, *SE* = 2.52) to Time 2 (*M* = 7.01, *SE* = 2.51). For the control group, the effect of time is not significant, *F*(1, 544) = 0.134, *p* = .714, indicating no change in speed (Time 1, *M* = 0.561, *SE* = 2.58, Time 2: *M* = 0.972, *SE* = 2.58). Baseline velocity at Time 1 was similar between the pramipexole group (*M* = 388°, *SD* = 70) and the control group (*M* = 381°, *SD* = 60), *t*(37) = 0.36, *p* = .71).

### Saccadic reaction time

While velocity measures energisation, Reaction Time may measure speed of decision processes (Ghez and Krakauer 2006; Haith et al. 2016). We next asked whether pramipexole also speeds up decisions. There were no drug effects. Reaction time was shorter where outcomes were uncertain (*M* = 203, *SE* = 4.84) compared to the noncontingent conditions where outcomes were certain (*M* = 209, *SE* = 4.84), (main effect of contingency, *F*(1, 210) = 6.58, *p* = .011), and when motivation was high (*M* = 202, *SD* = 4.84) compared to when motivation was low condition (*M* = 208, *SD* = 4.84) (main effect of motivation, *F*(1, 210) = 9.47, *p* = .011). There was also a practice effect, with shorter reaction time at Time 2 (*M* = 202, *SE* = 4.84) compared to Time 1 (Time 1: *M* = 209, *SE* = 4.84) (main effect of time: *F*(1, 210) = 15.8, *p* < .001), with an interaction between motivation and contingency (*F*(1, 303) = 11.1, *p* < .001; see supplemental Materials for post-hoc comparisons). However, none of the other fixed effects were significant (all *p*s > 0.05).

### Saccadic amplitude

Larger amplitudes of movement often accompany increases in speed. Although pramipexole increased velocity, it had no effect on the amplitude of movement (all *p* > .05 for drug effects). There was a significant interaction between motivation and contingency, *F*(1. 311) = 5.94, *p* = .015. To further examine this, LME models was fitted separately for the contingent conditions and non-contingent conditions, however none of the fixed effects within these separate models were significant (all *p*s > 0.05).

### Saccadic endpoint variability

To test whether the effect of pramipexole on vigour could was accompanied by a speed-accuracy trade-off, we examined end-point variability (S1). There was no drug effect on motor variability (Time x Group, *F*(1, 35) = 1.86, *p* = .182) suggesting pramipexole increased speed without trading accuracy. There was greater endpoint variability when outcome was certain (*M* = 1.06, *SD* = 0.067) compared to when reward outcome was uncertain (*M* = 0.868, *SD* = 0.052) (main effect of contingency (1, 35) = 25.9, *p* < .001, *np2* = 0.425), and when motivation was low (*M* = 1.06, *SD* = 0.065) compared to when motivation was high (*M* = 0.865, *SD* = 0.051) (main effect of motivation (1, 35) = 44.8, *p* < .001, *np2* = 0.561). There was also a significant interaction between contingency and motivation *F*(1, 35) = 25.0, *p* < .001, *np2* = 0.417 (see supplemental materials for post-hoc comparisons).

### Pupil dilatation

Previous studies (Satterthwaite et al. 2007; Chiew and Braver 2014) showed that motivation and uncertainty both cause pupil dilation, so we wanted to test if pramipexole affects this. Accordingly, pupil dilation was greater when outcomes were uncertain (*M* = 320, *SE* = 20.0) compared to when outcomes were certain (*M* = 297, *SE* = 19.9) (main effect of contingency, *F*(1, 187) = 6.11, *p* = .014), and when motivation was high (*M* = 219, *SD* = 19.9) compared to when motivation was low (*M* = 298, *SD* = 19.9) (main effect of motivation, *F*(1, 187) = 5.32, *p* = .022). Pramipexole did not have any effects on pupil (Time × Group interaction, (*F*(1, 194) = 1.58, *p* = .211), unlike on vigour. Contingent motivation caused greater pupil dilation than non-contingent motivation, in alignment with previous studies (contingency x motivation, *F*(1, 298) = 4.49, *p* = .035, see Supplemental Materials for post-hoc comparisons).

## Discussion

Here we examined whether pramipexole affects response vigour following cues predictive of reward in healthy adult participants. Saccadic vigour is sensitive to effects of reward, contingency and dopamine(Manohar et al. 2017; Grogan et al. 2020). In the current study, the dopamine agonist pramipexole increased motor vigour in general. Movements were faster when participants were motivated, and when they were on pramipexole (Fig. 3), However, this was independent of whether reward was expected or not (Win vs. Nothing), and whether reward was dependent on performance (Performance Vs Random). This pattern of findings suggests that pramipexole has a blanket effect on vigour, irrespective of extrinsic cues indicative of reward.

The lack of interaction between pramipexole and reward contingency suggests that “motor motivation” or intrinsic motivational vigour is under dopaminergic control that may be distinct from the effects of extrinsic reward (Mazzoni et al. 2007). A possible explanation for this is that pramipexole increases tonic dopaminergic activity, leading to a higher estimate of the average reward rate (Niv et al. 2007), which in turn invigorates action. Interestingly, Grogan et al. (Grogan et al. 2020) found that dopaminergic stimulation in patients with Parkinson’s Disease increased the effect of reward contingency on response vigour but weakened the effect of guaranteed reward. They proposed a dissociation of two dopaminergic pathways responsible for performance-based motivation and mere reward expectation. In their model, dopamine strengthens contingent motivation via the caudate nucleus and weakens motivation by expected reward via the nucleus accumbens and ventral pallidum in patients with Parkinson’s disease. The differences in the effect of dopamine between our study and the study by Grogan et al. could be attributable to the differences in the participant samples. The current study consisted of a healthy adult sample with no impairment in the dopaminergic system that, by default, has higher response vigour for contingent rewards compared to guaranteed reward (Manohar et al. 2017).

To assess whether the increase in saccadic velocity due to pramipexole action was not instead caused by a trade-off between speed and accuracy, we also examined saccadic amplitude and endpoint variability. We found that saccadic amplitude and endpoint variability were not compromised by increase in the speed of saccade, consistent with previous studies using the same paradigm (Manohar et al. 2017; Grogan et al. 2020).

In line with previous work (Manohar et al. 2017), pupil dilatation was increased with contingent reward, but not guaranteed reward, in healthy adults. In the current study, adding pramipexole did not increase pupil dilatation for either contingent nor guaranteed rewards. This suggests that treatment with pramipexole did not lead to a rise in autonomic arousal. There was also no effect of pramipexole on reaction times, demonstrating that the effect was specific to saccadic vigour. Together, these results suggest a primary effect of pramipexole on motor vigour as opposed to a secondary effect arising from reduction in tiredness or an increased generalised arousal, or faster decision-making. Vigour and saccadic reaction time were both affected by practice, such that reaction times were overall shorter in Time 2 compared to Time 1.

Our study has several limitations, including the lack of measures of baseline dopamine and plasma levels of pramipexole in participants and how these relate to performance on the saccade task. A power calculation was not performed before this study, however, effect sizes in previous work (0.61, Norbury et al. 2013; 0.49, Schuck et al. 2002; 0.64, Riba et al. 2008) are consistent with ours and a post-hoc power estimate suggests we had 75% power to detect an effect.

While participants were randomised and blinded to their group status, there may be a chance that participants could become aware of their group status due to experience of side effects, as the prampexole group reported more dizziness, somnolence, and nausea (see Supplemental Materials). Pill count and diary record were not to monitor medication adherence therefore it is unclear whether participants deviated from the titration schedule. The unblinded nature of the data analysis could have introduced bias into the results.

The 12 to 15 days study duration was selected to allow time for titration and to minimise adverse effects in healthy volunteers who otherwise would not have required pramipexole treatment. As far as the authors are aware, other human studies using pramipexole had either used a single dose in healthy volunteers (e.g. Gallant et al. 2016; Pizzagalli et al. 2008; Santesso et al. 2009) or much longer study durations in clinical populations (e.g. Tundo et al. 2019). It remains an important question whether our findings in healthy people might generalise to patients, such as people with depression or Parkinson’s Disease. It is possible that dopamine is contributing to the improvements seen in depression by generally increasing energy, despite not having selective impact on reward processing. It may suggest that the effects are non-selective and may link to the fact that prampexole is also implicated in impulse control disorders in some individuals. Further work is required to link the effect of the drug on task performance and its potential clinical effects on symptoms.

In conclusion, we found that the dopamine agonist pramipexole enhanced oculomotor response vigour in healthy adults, irrespective of cues predictive of contingent or expected reward, and it does so without compromising the accuracy of saccades. Given the reduced motivation characteristic of depressed, and particularly anhedonic patients, it would be interesting to assess in future work whether changes in motivational vigour mediate the clinical effects of this medication.

### Electronic supplementary material

Below is the link to the electronic supplementary material.

Supplementary Material 1
