# Supplementary material for: The effects of pramipexole on motivational vigour during a saccade task: a placebo-controlled study in healthy adults
Source: Psychopharmacology (Berl). 2024 Mar 18;241(7):1365–75. doi: 10.1007/s00213-024-06567-z (PMC11199222; doi:10.1007/s00213-024-06567-z)
Supplement: Supplementary file 1 — Supplementary Material 1 [file 213_2024_6567_MOESM1_ESM.docx]

**Supplemental Materials**

**Titration and Tapering Schedule for the current study**

The following dosing scheme was used with dosing occurring once a day in the evening

**Pramipexole salt**

**Initiation of treatment:**

Day 1: 0.25mg ***<< MEETING 2 (before drug is taken)***

Day 2: 0.25mg 🡨 ***CALL 1 (before the second dose of drug is taken in the evening)***

Day 3: 0.25mg

Day 4: 0.5mg

Day 5: 0.5mg

Day 6: 0.5mg

Day 7: 0.75mg

Day 8: 0.75mg 🡨 ***CALL 2***

Day 9: 0.75mg

**Peak maintenance:**

Day 10: 1.0mg

Day 11: 1.0mg

Day 12: 1.0mg ***<< TIME WINDOW FOR MEETING 3 AND 4***

Day 13: 1.0mg ***<< TIME WINDOW FOR MEETING 3 AND 4***

Day 14: 1.0mg ***<< TIME WINDOW FOR MEETING 3 AND 4***

Day 15: 1.0mg ***<< TIME WINDOW FOR MEETING 3 AND 4***

**Discontinuation of treatment:**

Day 16: 0.75mg

Day 17: 0.5mg

Day 18: 0.25mg

**Day after last dose has been taken** 🡨 ***CALL 3***

**Post-hoc Comparisons on Saccadic Reaction Time**

To further examine the motivation x contingency interaction, LME models were fitted separately for the contingent conditions and non-contingent conditions. Reaction time was significantly slower when reward was not expected (Nothing condition: *M* = 212, *SD* = 5.10) compared to when reward was expected (Win Condition: *M* = 203, *SD* = 5.11) (main effect of motivation in the non-contingent condition: *F*(1, 97.8) =14.1, *p* = < .001). In contrast, reaction time did not differ between when reward was contingent on performance (*M* = 202, *SD* = 4.78) and when it was random (*M* = 204, *SD* = 4.78), (main effect of motivation in the contingent condition: *F*(1 , 96.6) = .467, *p* = .496).

**Post-hoc Comparisons on Saccadic Endpoint Variability**

To further investigate the interaction between contingency and motivation, two post hoc t-test were computed collapsing across time separately for the contingent conditions (Performance vs Random) comparison and the non-contingent (Win vs Nothing) comparison. Endpoint variability did not differ between when reward was contingent on performance condition (*M* = .852, *SD* = .301) and when reward was random (*M* = .887, *SD* = .341), whereas endpoint variability was significant greater when reward was not expected (Nothing Condition: *M* = 1.24, *SD* = .509) compared to when reward was expected (Win condition: *M* = .881, *SD* = 352) (see Figure 4).

None-Contingent Comparison

Contingent Comparison

Figure 4. Endpoint Variability Means (Error Bars represents +SEM).

**Post-hoc Comparisons on Pupil Dilatation**

Separate LMEs were computed for the contingent conditions and the non-contingent conditions. These showed greater dilation when reward was based on performance (*M* = 339, *SE* = 21.1) than reward was random (*M* = 304, *SE* = 21.1) (contingent conditions; fixed effect of motivation, *F*(1, 92.7) = 7.37, *p* = .008), but pupil dilation did not differ between when reward was expected (Win Condition: *M* = 299, *SE* = 20.4) when reward was not expected (Nothing Condition: (*M* = 291, *SE* = 20.3). (non-contingent conditions: *F*(1, 88.8) = .444, *p* = .507).

**Side effects experienced by the two drug groups**

|  | Control (*n* = 18) | Pramipexole (*n* = 19) |  |
| --- | --- | --- | --- |
| Side effects Count* | Group Sum | Group Sum | Fisher’s Exact |
| Sleeping problems | 4 | 7 | .271 |
| Abnormal dreaming | 4 | 4 | .621 |
| Headache | 4 | 5 | .538 |
| Dizziness | 1 | 7 | .025* |
| Somnolence | 3 | 9 | .038* |
| Nausea | 1 | 13 | <.001* |
| Vomiting | 0 | 4 | .059 |
| Constipation | 0 | 1 | .514 |
| Fatigue | 3 | 5 | .379 |
| Impulse control problems | 1 | 2 | .521 |
| Hallucination | 0 | 0 | - |
| Abnormal movements | 0 | 1 | .514 |

*side effect count is the number of participants who reported experiencing side effects (either mild, moderate or severe). Proportions of participant with and without side effects are compared between group using Fisher’s Exact test (1-sided) as we would expect participants on active medication to be more likely to experience side effects.
